# The role of enterocyte defects in the pathogenesis of congenital diarrheal disorders

**DOI:** 10.1242/dmm.022269

**Published:** 2016-01-01

**Authors:** Arend W. Overeem, Carsten Posovszky, Edmond H. M. M. Rings, Ben N. G. Giepmans, Sven C. D. van IJzendoorn

**Affiliations:** 1 Department of Cell Biology, University Medical Center Groningen, University of Groningen, 9713 AV Groningen, The Netherlands; 2 Department of Pediatrics and Adolescent Medicine, University Medical Center Ulm, 89075 Ulm, Germany; 3 Department of Pediatrics, Erasmus Medical Center Rotterdam, Erasmus University Rotterdam, 3000 CB Rotterdam, The Netherlands; 4 Department of Pediatrics, Leiden University Medical Center, Leiden University, 2300 RC Leiden, The Netherlands

**Keywords:** Brush border, Cell polarity, Congenital diarrheal disorder, Enterocyte, Intracellular trafficking, Microvillus inclusion diseases

## Abstract

Congenital diarrheal disorders are rare, often fatal, diseases that are difficult to diagnose (often requiring biopsies) and that manifest in the first few weeks of life as chronic diarrhea and the malabsorption of nutrients. The etiology of congenital diarrheal disorders is diverse, but several are associated with defects in the predominant intestinal epithelial cell type, enterocytes. These particular congenital diarrheal disorders (CDD^ENT^) include microvillus inclusion disease and congenital tufting enteropathy, and can feature in other diseases, such as hemophagocytic lymphohistiocytosis type 5 and trichohepatoenteric syndrome. Treatment options for most of these disorders are limited and an improved understanding of their molecular bases could help to drive the development of better therapies. Recently, mutations in genes that are involved in normal intestinal epithelial physiology have been associated with different CDD^ENT^. Here, we review recent progress in understanding the cellular mechanisms of CDD^ENT^. We highlight the potential of animal models and patient-specific stem-cell-based organoid cultures, as well as patient registries, to integrate basic and clinical research, with the aim of clarifying the pathogenesis of CDD^ENT^ and expediting the discovery of novel therapeutic strategies.

## Abstract (precis)

**Summary:** Overview of the recent progress in our understanding of congenital diarrheal disorders, and the available models to study these diseases.

## Introduction

Congenital diarrheal disorders (CDDs) are a group of rare inherited intestinal disorders that are characterized by persistent life-threatening intractable diarrhea and nutrient malabsorption, which emerge during the first weeks of life. The etiology of CDDs is diverse, including defects in enteroendocrine cells, dysregulation of the intestinal immune response, or defects in the predominant cell type of the intestinal epithelium, the enterocyte (Canani et al., 2015).

CDDs associated with enterocyte defects (abbreviated as CDD^ENT^) include disorders that can be treated with nutrition therapy. Other CDD^ENT^ require life-long total parenteral nutrition (TPN; see Box 1 for a glossary of clinical terms used in this article) to receive adequate nutrition, and are a leading indication for pediatric intestinal transplantations (Halac et al., 2011). Most CDD^ENT^ are difficult to diagnose, and clinical management is restricted to the treatment of symptoms; there is currently no cure. If left untreated, CDD^ENT^ are invariably fatal.

**Box 1. Clinical glossary**

**Aminoaciduria:** a disorder of protein metabolism in which excessive amounts of amino acids are excreted in the urine.

**Atresia:** the congenital absence, or the pathological closure, of an opening, passage, or cavity.

**Bowel rest:** the intentional restriction of oral nutrition.

**Chronic diarrhea:** the passage of three or more loose or liquid stools per day for more than 2-4 weeks.

**Hepatomegaly:** enlargement of the liver.

**Hypercalciuria:** the presence of abnormally high levels of calcium in the urine; usually the result of excessive bone loss in hyperparathyroidism or osteoporosis.

**Hypobetalipoproteinemia:** a hereditary disorder characterized by low levels of beta-lipoproteins, lipids and cholesterol.

**Hypocholesterolemia:** the presence of abnormally small amounts of cholesterol in the circulating blood.

**Intractable diarrhea:** treatment-resistant, non-infectious diarrhea with high mortality; need for total parenteral nutrition. Intractable diarrhea of infancy is a heterogeneous syndrome with different etiology.

**Intrahepatic cholestasis:** obstruction within the liver that causes bile salts, bile pigments and lipids to accumulate in the bloodstream.

**Metabolic acidosis:** a clinical disturbance characterized by an increase in plasma acidity.

**Punctate keratitis:** a condition characterized by a breakdown or damage of the epithelium of the cornea in a pinpoint pattern.

**Siderosis:** a form of pneumoconiosis due to the inhalation of iron particles.

**Total parenteral nutrition (TPN):** intravenous feeding that provides patients with all the fluid and the essential nutrients they need when feeding by mouth is inhibited.

**Trichothiodystrophy:** an autosomal recessive inherited disorder characterized by brittle hair and intellectual impairment.

**Woolly hair:** unusually curled hair.

Owing to the consanguinity of parents of affected children, genetic defects associated with CDD^ENT^ have recently been identified. The clinical consequences of some mutations – those affecting specific transporter proteins or certain enzymes – are relatively straightforward, such as in individuals with congenital lactase deficiency or sucrase-isomaltase deficiency caused by loss-of-function mutations in lactase (Behrendt et al., 2009; Kuokkanen et al., 2006) and sucrase-isomaltase (Ritz et al., 2003), respectively. Other mutations, however, are in genes that have less well-understood functions in intestinal epithelial physiology, such as in individuals with microvillus inclusion disease (MVID), congenital tufting enteropathy (CTE), familial hemophagocytic lymphohistiocytosis type 5 (FHL5) and trichohepatoenteric syndrome (THES) (Fabre et al., 2012; Hartley et al., 2010; Heinz-Erian et al., 2009; Müller et al., 2008; Sivagnanam et al., 2008; Szperl et al., 2011; Wiegerinck et al., 2014; zur Stadt et al., 2009). Table 1 summarizes CDD^ENT^-associated genes, the proteins they encode and their function. Understanding the mechanisms by which these mutations lead to disease should pinpoint targets for improved diagnosis and therapeutic intervention.

**Table 1.**
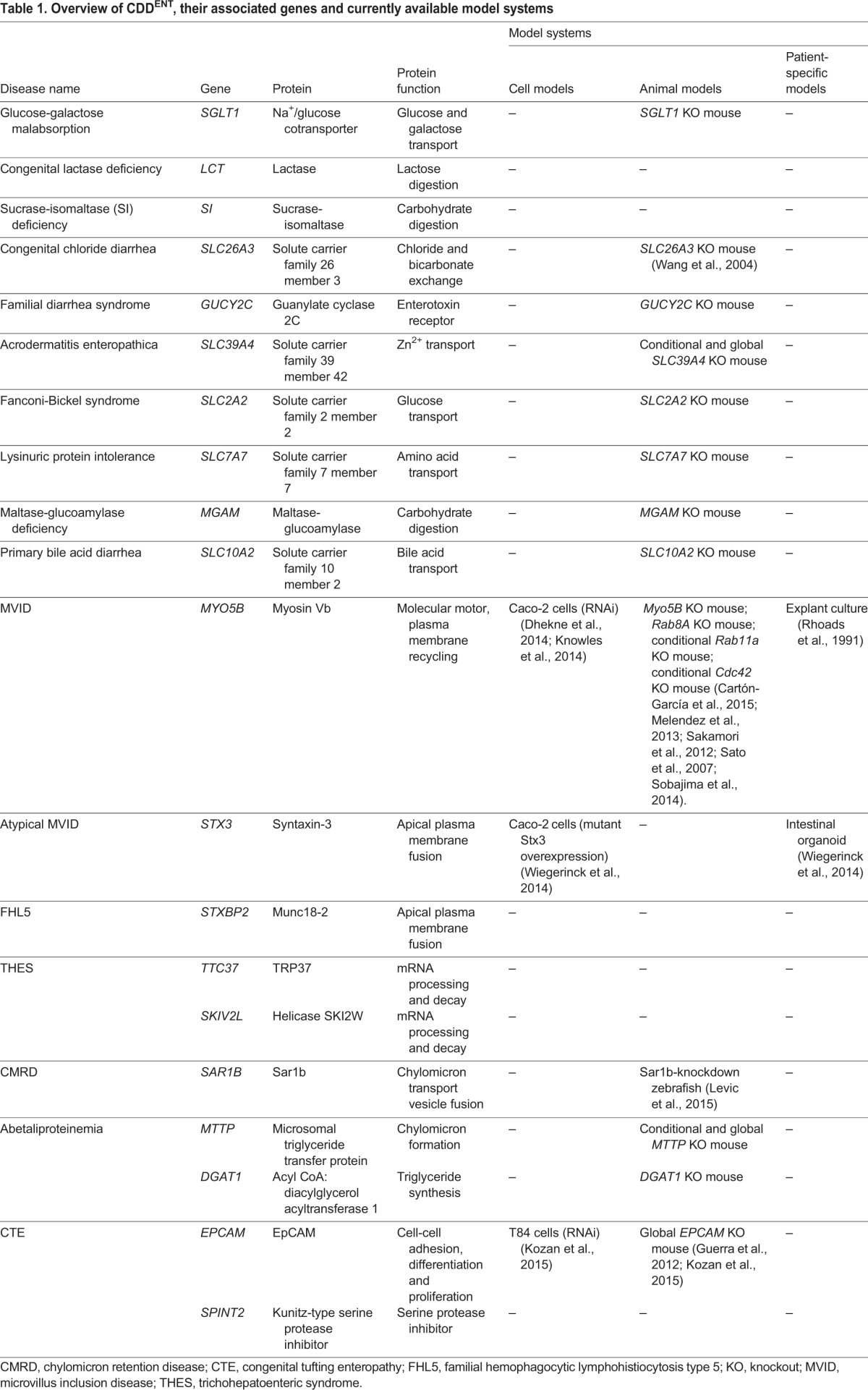
**Overview of CDD^ENT^, their associated genes and currently available model systems**

The identification of genetic mutations in individuals with CDD^ENT^ has confirmed the autosomal recessive inheritance pattern of these diseases; thus, genetic counselling and prenatal diagnosis are important tools for heterozygote carriers. Because the histological hallmarks that characterize some CDD^ENT^ can be very subtle and easily missed, the identification of genetic defects contributes to a better and faster differential diagnosis, which is currently offered by several medical centers worldwide.

Here, we discuss the different CDD^ENT^, recent discoveries concerning their underlying molecular and genetic mechanisms, and the model systems used in researching these disorders. Further, basic research is urgently needed to improve the diagnosis and management of these devastating diseases, and for developing new therapeutic strategies to combat them.

## Enterocytes: a brief overview

**Fig. 1.**
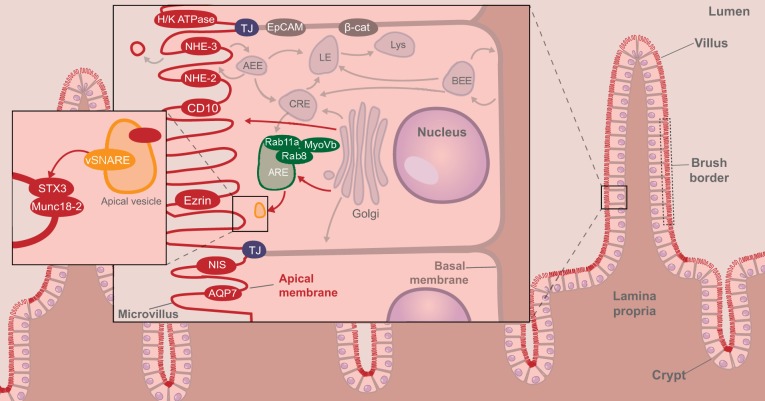
**Schematic overview of tissue and cellular characteristics of healthy intestinal epithelium and enterocytes.** In healthy enterocytes, the apical recycling endosome (ARE; green) is located sub-apically, and is important for transporting apically residing proteins (depicted in red) to the apical membrane (brush border), via mechanisms that are not well understood that involve the small GTPases Rab11a and Rab8, and the effector protein myosin Vb (MyoVb; see text). At the apical membrane, syntaxin-3 (STX3) and Munc18-2 (*STXBP2*) are involved in the fusion of the membrane-bound apical vesicle (orange). β-catenin (β-cat) and EpCAM mediate cell-cell adhesion. Other organelles and common trafficking routes are shown in light gray (not discussed here). AEE, apical early endosome; AQP7, aquaporin-7; BEE, basolateral early endosome; CRE, common recycling endosome; LE, late endosome; Lys, lysosome; NHE, sodium/hydrogen exchanger; NIS, Na/I symporter; TJ, tight junction.

Enterocytes are the absorptive cells in the lining of the intestinal mucosa. Enterocytes originate from the intestinal stem cells that reside in the intestinal crypts (Sato et al., 2009), and differentiate and migrate within 3-4 days from the crypt to the villus tip, where they are extruded into the gut lumen. Enterocytes are arranged as a monolayer of polarized epithelial cells (Fig. 1) (Massey-Harroche, 2000). Their plasma membrane consists of a basal and a lateral domain, facing the underlying tissue and neighboring cells, respectively, and an apical domain, facing the gut lumen. Densely packed microvilli, supported by an actin filament meshwork, protrude from the apical surface, resulting in a brush border appearance. Microvilli increase the absorptive surface area of the cells and release small vesicles that contribute to epithelial-microbial interactions (Crawley et al., 2014; Shifrin et al., 2012). The plasma membrane domains are equipped with distinct enzymes and transporter proteins that control the metabolism, absorption and/or secretion of nutrients, metabolites and electrolytes between the gut lumen, cell interior and body tissue. The polarized distribution of these proteins at the different plasma membrane domains is secured by their intracellular sorting and trafficking via the Golgi apparatus and endosomes (van der Wouden et al., 2003; Weisz and Rodriguez-Boulan, 2009). Tight junctions between the apical domain and the lateral surface domain provide tight intercellular adhesion, which limits protein diffusion between the apical and lateral plasma membrane domains, and controls the paracellular transport of electrolytes and water (Giepmans and van Ijzendoorn, 2009; Marchiando et al., 2010). Adherens junctions in the lateral domain mediate cell-cell adhesion strength (Giepmans and van Ijzendoorn, 2009). Enterocyte polarity and cell-cell adhesion junctions together provide the selectively permeable barrier function of the intestinal epithelial monolayer.

## Diverse molecular mechanisms of CDD^ENT^ underlying clinical presentation and diagnosis

Based on recent molecular and cell biological studies, enterocyte defects that underlie CDD^ENT^ can be divided into defects of (i) brush-border-associated enzymes and transporter proteins; (ii) intracellular protein transport; (iii) intracellular lipid transport and metabolism; and (iv) intestinal barrier function (Table 1).

## Defects of brush-border-associated enzymes and transporter proteins

The majority of CDD^ENT^ are caused by autosomal recessive mutations in genes that encode brush-border-associated enzymes and transporter proteins (Canani et al., 2015) (Table 1). Depending on the type of mutation, these proteins are either not expressed, not correctly transported to the brush border membrane, or display defects in their activity, resulting in defective digestion, absorption and/or transport of nutrients, metabolites and/or electrolytes at the enterocyte brush border. Subsequent changes in the concentration of osmotically active compounds in the gut lumen cause diarrhea. Prototypical examples of these CDD^ENT^ are glucose-galactose malabsorption (caused by mutations in the Na^+^/glucose cotransporter gene, *SGLT1*) (Martín et al., 1996), congenital lactase deficiency (mutations in the lactase gene, *LCT*) (Kuokkanen et al., 2006), sucrase-isomaltase (SI) deficiency (caused by mutations in the *SI* gene) (Ritz et al., 2003), congenital chloride diarrhea (caused by mutations in the solute carrier family 26 member 3 gene, *SLC26A3*) (Wedenoja et al., 2011); several other CDD^ENT^ can also be included in this category (Canani and Terrin, 2011) (Table 1).

Individuals with familial diarrhea syndrome have activating mutations in *GLUCY2C*, which encodes the guanylate cyclase 2C protein. Mutated guanylate cyclase 2C enhances cellular cGMP levels (Fiskerstrand et al., 2012). cGMP stimulates cystic fibrosis transmembrane conductance regulator (CFTR) activity in the brush border of enterocytes by stimulating its proper translocation, resulting in enhanced secretion of chloride and water (Golin-Bisello et al., 2005). CDD^ENT^ associated with functional defects of brush-border-associated enzymes and transporter proteins are typically not associated with abnormal enterocyte organization, as examined by histology.

## Defects in intracellular protein transport

In other CDD^ENT^, apical brush-border-associated enzymes and transporter proteins are collectively mislocalized in the enterocytes, indicative of general defects in intracellular protein transport. Examples of CDD^ENT^ characterized by this class of defect are described below.

### Microvillus inclusion disease

Individuals with MVID suffer from persistent diarrhea, nutrient malabsorption and failure to thrive (Cutz et al., 1989). In most cases (95%), symptoms develop within days after birth, but a late-onset variant, which manifests 2-3 months postnatally, has also been described (Cutz et al., 1989). Variable extra-intestinal symptoms include intrahepatic cholestasis and renal Fanconi syndrome (van der Velde et al., 2013) (see MVID case study in Box 2). Some individuals with MVID present less-severe digestive symptoms for reasons that are not clear (Perry et al., 2014).

**Box 2. Case study: MVID presenting with renal Fanconi syndrome**

A boy born to unrelated parents, born at term by spontaneous vaginal delivery after an uncomplicated pregnancy, was hospitalized 2 months after birth because of dehydration, metabolic acidosis, feeding intolerance and intractable diarrhea. The diarrhea persisted during fasting and showed elevated stool sodium content consistent with secretory diarrhea. He was given total parenteral nutrition (TPN) via a central venous line. Exhaustive etiological investigations ruled out infectious or allergic etiologies. Duodenum biopsies were taken and processed for light microscopy and electron microscopy (EM) examination. A moderate degree of villus atrophy, and partial intracellular periodic acid Schiff (PAS) and CD10 staining were observed. EM revealed moderate brush border atrophy but it took three rounds of examination before microvillus inclusions were found, and the diagnosis of MVID was accordingly made. The patient was discharged on home TPN. When hospitalized for the evaluation of growth failure, excessive urinary losses of phosphate were observed without rapid catch-up of weight gain. Examination showed severely reduced tubular phosphate resorption, hypercalciuria, generalized aminoaciduria and severe rickets, which are characteristics of renal Fanconi syndrome. No disturbances in glomerular function were observed. Phosphorus in the parenteral nutrition was increased stepwise and treatment with oral phosphate was added. The parenteral and oral supplementation of phosphate resulted in a gradual increase in serum phosphate levels, a decrease of alkaline phosphatase, a normalization of the bone density and resolution of his rickets. Also, catch-up growth was obtained. Laboratory results indicated that the persistence of renal Fanconi syndrome gradually resolved after the patient received a multi-organ transplant (small intestine, large intestine, pancreas and liver) at the age of 5 years, and enteral feeding was fully restored. Examination of kidney biopsies from this patient revealed no intracellular PAS staining in the proximal tubular epithelial cells and, at the ultrastructural level, proximal tubular epithelial cells showed a normal apical brush border. This patient illustrates the clinical complications and underscores the need for reliable genotype-phenotype correlations to understand the extra-intestinal clinical symptoms.

MVID, which is diagnosed by intestinal biopsy, features villus atrophy, microvillus atrophy, and the redistribution of CD10 and periodic acid Schiff (PAS)-stained material from the brush border to intracellular sites (Phillips et al., 2000) in the enterocytes. Staining of the epithelial cell-cell adhesion protein EpCAM, aberrant in CTE, is normal (Martin et al., 2014). A definitive diagnosis is recommended prior to potential intestinal transplantation, and this includes analysis by electron microscopy (EM) for microvillus inclusions in the cytoplasm of enterocytes. The frequency of such inclusions can be very low and repeated rounds of EM analyses can be required, although semi-automated EM might help to increase the efficiency of screening (de Boer et al., 2015). Immuno-based detection of villin, which marks microvillus inclusions, has been proposed to be a useful adjunct in MVID diagnosis (Shillingford et al., 2015). Notably, microvillus inclusions are also present in rectal biopsies, facilitating diagnosis if a duodenal biopsy is not feasible. Some individuals with clinical symptoms typical of MVID show no microvillus inclusions but do show the other enterocyte abnormalities, suggesting that MVID is a heterogeneous disease (Mierau et al., 2001).

**Fig. 2.**
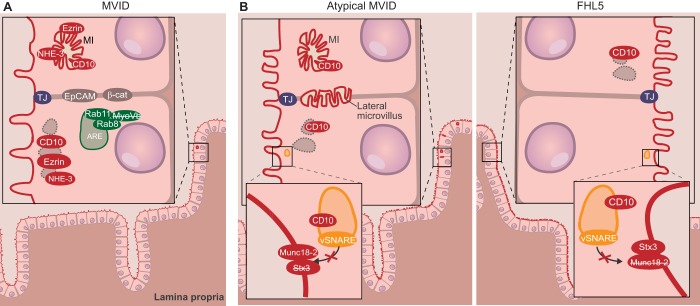
**Schematic overview of tissue and cellular defects associated with MVID and FHL5.** (A) In typical microvillus inclusion disease (MVID), which is caused by loss of MyoVb in the apical recycling endosome (ARE; green), villi are shortened, and microvilli are shortened and fewer in number (see Fig. 1 for comparison). The normally apically localized proteins Ezrin, NHE-3 and CD10 are mislocalized in microvillus inclusions (MIs) or in unknown intracellular compartments (gray, dotted lines). The ARE is localized near the nucleus instead of sub-apically. (B) In familial hemophagocytic lymphohistiocytosis type 5 (FHL5; right-hand panel), microvilli are shortened, whereas, in atypical MVID (left-hand panel), microvilli are both shortened and fewer in number. Loss of syntaxin-3 (STX3), as occurs in atypical MVID, or of Munc18-2, as occurs in FHL5, inhibits the fusion of vesicles with the apical membrane, resulting in the intracellular retention of apical proteins (demonstrated here for CD10). Additionally, the formation of MIs and of lateral microvilli occurs in atypical MVID, but not in FHL5. β-cat, β-catenin; MyoVb, myosin Vb; NHE, sodium/hydrogen exchanger; STX3, syntaxin-3; TJ, tight junction.

MVID and variants of MVID are associated with *MYO5B*, *STXBP2* and *STX3* mutations (Table 1) (Müller et al., 2008; Ruemmele et al., 2010; Stepensky et al., 2013; Szperl et al., 2011; Wiegerinck et al., 2014). Deletion of the *Myo5B* gene in mice causes the development of early-onset MVID (Cartón-García et al., 2015). *MYO5B* encodes the actin-based motor protein myosin Vb, which consists of an N-terminal actin-binding motor domain and a C-terminal tail domain that includes the cargo-binding domain. Based on crystal structures of the myosin Vb protein, mutations in *MYO5B* have been functionally categorized (van der Velde et al., 2013). The myosin Vb cargo-binding domain binds selectively to small Rab GTPases, including RAB11A and RAB8A. Myosin Vb, RAB11A and RAB8A associate with apical recycling endosomes (AREs) in polarized epithelial cells, where they control the activity of the small GTPase CDC42 (Bryant et al., 2010), and both myosin Vb and RAB11A are mislocalized in MVID enterocytes (Fig. 2) (Dhekne et al., 2014; Szperl et al., 2011). Accordingly, in addition to *Myo5B* knockout (KO) mice (Cartón-García et al., 2015), mice in which the intestinal *Rab8a*, *Rab11a* or *Cdc42* genes have been individually deleted also develop the cellular hallmarks of MVID (Melendez et al., 2013; Sakamori et al., 2012; Sato et al., 2007; Sobajima et al., 2014). However, diarrhea is not observed in *Rab11a* or *Cdc42* KO mice, and *Rab8a* KO mice survive for approximately 5 weeks after birth, thus more closely resembling the phenotype of late-onset MVID. Mutagenesis of residues in myosin Vb that mediate this protein's interaction with either RAB11A or RAB8A, and the subsequent introduction of these mutant forms into myosin Vb-silenced human Caco-2 cells (Caucasian colon adenocarcinoma), revealed that the uncoupling of myosin Vb from both RAB11A and RAB8A forms the basis of MVID pathogenesis (Knowles et al., 2014).

Rab11a- and Rab8a-positive AREs play a pivotal role in epithelial polarity development (Bryant et al., 2010; Golachowska et al., 2010; Overeem et al., 2015; Wakabayashi et al., 2005). Rab11a-positive AREs localize in close proximity to the apical brush border surface in enterocytes and harbor signaling molecules, including: phosphoinositide-dependent protein kinase-1 (PDK1) (Dhekne et al., 2014; Kravtsov et al., 2014); the PDK1 target, atypical protein kinase C-iota; and the ezrin-phosphorylating kinase, Mst4 (Dhekne et al., 2014). Myosin Vb is required for the polarized, subapical localization of Rab11a-positive AREs (Szperl et al., 2011), which, in turn, is required for efficient Mst4-mediated phosphorylation of ezrin and for ezrin-controlled microvilli development (Dhekne et al., 2014). Myosin-Vb-controlled AREs might thus function as a subapical signaling platform that regulates the absorptive surface area of enterocytes (Dhekne et al., 2014). Interestingly, ezrin depletion in the mouse intestine leads to a disorganized subapical actin filament web and causes microvillus atrophy (Saotome et al., 2004), similar to that seen in individuals with MVID. The presence of ezrin at the intestinal brush border correlates with the expression and function of the Na^+^/H^+^ hydrogen exchanger (NHE)-3, which regulates sodium absorption, and loss of Nhe-3 in mice leads to diarrhea (Ledoussal et al., 2001). MVID enterocytes show reduced NHE-3 expression (Ameen and Salas, 2000), and MVID jejunal explants revealed a net secretory state of the jejunum (Rhoads et al., 1991).

The ectopic expression of the myosin Vb tail domain, which acts as a dominant-negative mutant by competing with endogenous myosin Vb for the Rab proteins, can disrupt the delivery of proteins from Rab11a-positive AREs to the apical plasma membrane (Golachowska et al., 2010). The mechanism by which myosin Vb controls apical-surface-directed transport of proteins from AREs is not fully understood. Interestingly, individuals with mutations in either *STX3* (Wiegerinck et al., 2014), which encodes the transmembrane protein syntaxin-3, or *STXBP2* (Stepensky et al., 2013), which encodes Munc18-2, develop the clinical symptoms and cellular characteristics of MVID. Mutations in *STX3* or *STXBP2* give rise to disorders termed atypical MVID and FHL5, respectively (Fig. 2). In enterocytes, syntaxin-3 resides at the apical cell-surface domain, where it, in concert with SNAP23 and Munc18-2, mediates the fusion of transport vesicles with the apical plasma membrane (Riento et al., 2000). MVID-associated *STX3* mutations cause the depletion of syntaxin-3 or the expression of a syntaxin-3 protein that lacks the transmembrane domain (Wiegerinck et al., 2014), disrupting its function. *STXBP2* mutations abolish the interaction of Munc18-2 with syntaxin proteins (zur Stadt et al., 2009). Interestingly, enterocytes of conditional *Rab11* KO mice show altered localization of syntaxin-3 (Knowles et al., 2015). It is possible that myosin Vb mediates the apical trafficking of syntaxin-3 via AREs, and protein delivery to the apical cell surface. However, the effect of myosin Vb mutations on the apical membrane fusion machinery in MVID remains to be demonstrated. It should be noted that a homozygous mutation in *STX3* was also reported in an individual with autosomal recessive congenital cataracts and intellectual disability phenotype, without mention of intestinal symptoms (Chograni et al., 2015); thus, further investigation into genotype-phenotype correlation of the different *STX3* mutations is warranted.

Taken together, the available data suggest that defects in ARE function result in brush border microvillus atrophy and in the intracellular retention of enzymes and transporters that are required for the absorption of nutrients and ions by villus enterocytes, leading to the clinical phenotype of malabsorption and diarrhea in MVID (Dhekne et al., 2014; Knowles et al., 2014) (Fig. 2).

### Trichohepatoenteric syndrome

Individuals with THES present with intractable diarrhea in the first months of life accompanied by nutrient malabsorption and failure to thrive (Hartley et al., 2010). THES is associated with facial dysmorphism, hair abnormalities and, in some cases, skin abnormalities and immune disorders (Goulet et al., 2008). Some individuals with THES display trichothiodystrophy, liver disease, hepatomegaly and siderosis (see Box 1). Affected individuals are prone to infections, might fail to produce antibodies upon vaccination, or present with low immunoglobulin levels. Mild intellectual deficiency is a feature of ∼50% of all cases. THES can present as very-early-onset inflammatory bowel disease (Kammermeier et al., 2014). It is diagnosed on the basis of its clinical features and via biopsies of the small intestine, which reveal villus atrophy, variable immune cell infiltration of the thin layer of loose connective tissue that lies beneath the epithelium (called the lamina propria), and no specific histological abnormalities of the epithelium.

**Fig. 3.**
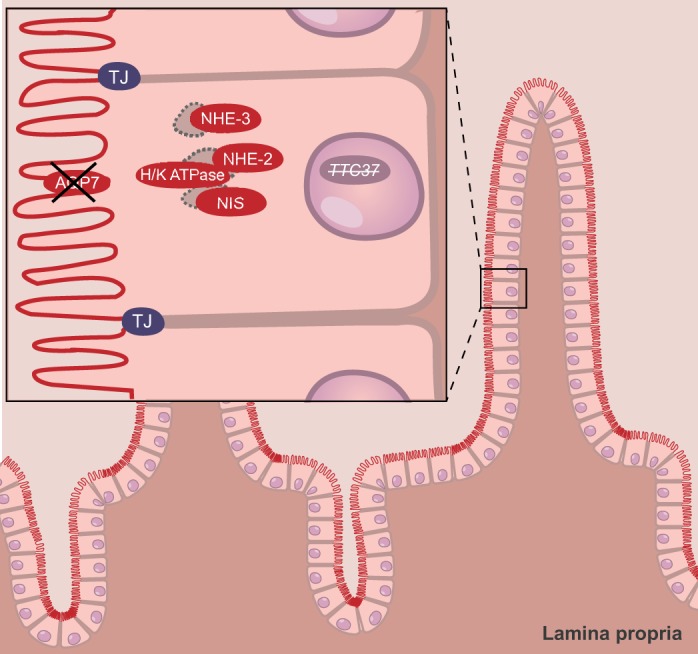
**Schematic overview of tissue and cellular defects associated with THES.** In individuals with trichohepatoenteric syndrome (THES), villus or microvillus defects are not observed. Through an unknown mechanism, loss-of-function mutations in *TTC37* result in the intracellular localization of the normally apically localized H^+^/K^+^-ATPase, the Na/I symporter (NIS), and the apical proteins NHE-2 and NHE-3. These mutations also result in the loss of expression, either global or local, of certain apical proteins, such as aquaporin-7 (AQP7). NHE, sodium/hydrogen exchanger; TJ, tight junction.

THES is associated with *TTC37* or *SKIV2L* mutations. *TTC37* encodes the tetratricopeptide repeat protein 37. *SKIV2L* encodes SKI2 homolog, superkiller viralicidic activity 2-like protein, which might be involved in antiviral activity by blocking translation of poly(A)-deficient mRNAs. In enterocytes with *TTC37* mutations, the brush-border-associated NHE-2 and -3, aquaporin-7, the Na^+^/I^−^ symporter, and the H^+^/K^+^-ATPase show reduced expression or mislocalization to the apical cytoplasm, with different patterns of mislocalization relative to their normal pattern (Hartley et al., 2010). NHE-2 and NHE-3 play an important role in salt and water absorption from the intestinal tract, and loss of *Nhe3* in the mouse intestine causes mild diarrhea (Ledoussal et al., 2001). In THES enterocytes, the brush border appears normal at the ultra-structural level, as does the basolateral localization of Na^+^/K^+^-ATPase (Hartley et al., 2010). Loss of TTC37 results in the defective trafficking and/or decreased expression of apical transport proteins, including aquaporin-7 (Fig. 3). The expression and distribution of apical transporters have not yet been analyzed for individuals with THES with *SKIV2L* mutations. The gene products of both *TTC37* and *SKIV2L* are human homologs of components of the yeast Ski complex, which is involved in exosome-mediated degradation of aberrant mRNA and associates with transcriptionally active genes (Fabre et al., 2012). *TTC37*, but not *SKIV2L*, is highly co-expressed with two genes involved in apical trafficking (*SCAMP1* and *EXOC4*; http://coxpresdb.jp/cgi-bin/coex_list.cgi?gene=9652&sp=Hsa2). The mechanism underlying THES is currently unknown, so further studies are needed to elucidate potential relationships between TTC37/SKIV2L, the Ski complex and the trafficking of apical transporter proteins.

Interestingly, another tetratricopeptide repeat protein, TTC7A, is implicated in a different disorder: multiple intestinal atresia (MIA). Stem-cell-derived intestinal organoids from a MIA individual show enterocyte polarity defects that are rescued by pharmacological inhibition of the small GTPase RhoA (Bigorgne et al., 2014; Overeem et al., 2015). Although MIA is not a CDD, these findings further accentuate the role of tetratricopeptide-repeat proteins in functional enterocyte polarity and associated intestinal disorders.

## Defects in intracellular lipid transport and metabolism

**Fig. 4.**
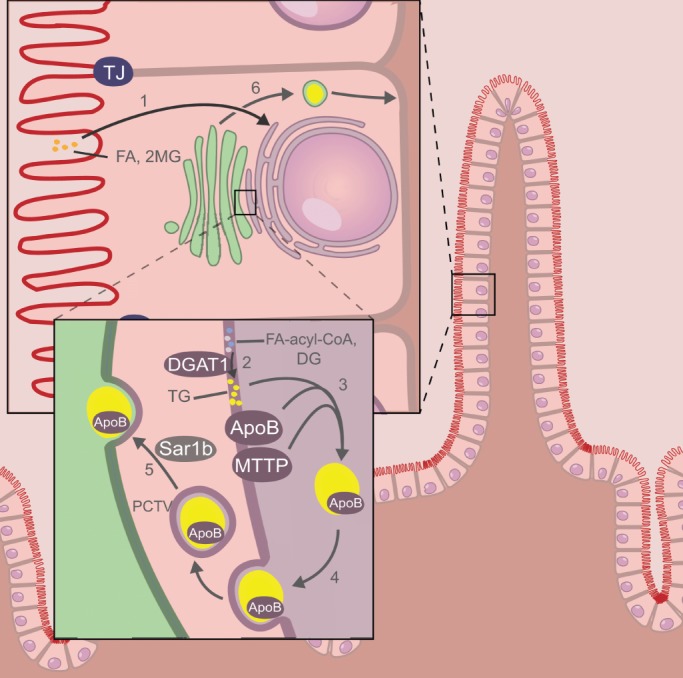
**Schematic overview of the cellular processes involved in lipid transport and metabolism** **in enterocytes.** After uptake from the lumen, fatty acids (FAs) and monoacylglycerol (2MG) are transported to the endoplasmic reticulum (ER) (1). Here (see magnified view), they are converted to triglycerides (TGs) in several metabolic steps (not shown), the last of which is dependent on DGAT1 (2). ApoB and MTTP act in concert to incorporate triglycerides into a chylomicron (yellow) (3). The newly formed chylomicron buds from the ER in a prechylomicron transport vesicle (PCTV) (4), which subsequently fuses with the Golgi, a process that is dependent on Sar1b (5). The chylomicron is then transported in a vesicle to the basal membrane, where it exits the cell (6). FA, fatty acid; 2MG, sn-2-monoacylglycerol; CoA, coenzyme A; DG, diacylglycerol; MTTP, microsomal triglyceride transfer protein; PCTV, prechylomicron transport vesicle; TG, triglyceride; TJ, tight junction.

In addition to defects in the intracellular transport of proteins, defects in the intracellular transport of lipids, summarized in Fig. 4, have been associated with CDDs. Our current understanding of the molecular mechanisms underlying this class of CDDs is summarized below.

### Chylomicron retention disease

Individuals with chylomicron retention disease (CMRD) suffer from chronic diarrhea, severe lipid malabsorption, failure to thrive, and hypocholesterolemia as a result of by hypobetalipoproteinemia. Large lipid vacuoles and chylomicron-like particles retained within membrane-bound compartments, which could represent pre-chylomicron transport vesicles, are typically observed in the cytoplasm of CMRD enterocytes. Microvilli appear normal by EM examination (Mouzaki et al., 2014).

CMRD is caused by mutations in *SAR1B* (Jones et al., 2003)*.* The Sar1b protein is part of the Sar1-ADP-ribosylation factor family of small GTPases and triggers the formation of coat protein complex II (COPII)-coated transport vesicles from the endoplasmic reticulum (Fig. 4). In CMRD, *SAR1B* mutations result in defective trafficking of nascent chylomicrons in pre-chylomicron transport vesicles between the endoplasmic reticulum and the Golgi apparatus, thereby interfering with the successful assembly of chylomicrons and their delivery to the lamina propria (Mansbach and Siddiqi, 2010). It remains unclear how defective intracellular chylomicron trafficking results in intestinal lipid malabsorption and diarrhea. Sar1 proteins are also involved in the trafficking of CFTR (Wang et al., 2004), which is a typical brush border protein in enterocytes. In the fruit fly *Drosophila melanogaster*, Sar1b is involved in the trafficking of Crumbs (Kumichel et al., 2015), a protein that controls apical-basal epithelial cell polarity also in the intestine (Whiteman et al., 2014). Whether *SAR1B* mutations in CMRD also affect the trafficking of apical brush border proteins in enterocytes and thereby contribute to impaired (lipid) absorption remains to be investigated.

### Familial hypobetalipoproteinemia and abetaliproteinemia

Two other CDD^ENT^ have been associated with defects in intestinal fat absorption and chylomicron assembly. Familial hypobetalipoproteinemia (FHBL), the only CDD^ENT^ that is dominantly inherited, is associated with mutations in the *APOB* gene, encoding apolipoprotein B (Young et al., 1990), which, together with triglycerides and other lipids, makes up the nascent chylomicron (Fig. 4). Abetaliproteinemia is associated with mutations in the *MTTP* gene, which encodes microsomal triglyceride transfer protein (MTTP). MTTP catalyzes the transfer of triglycerides to nascent ApoB particles in the endoplasmic reticulum. Abetaliproteinemia-associated mutations reduce MTTP activity, the synthesis of very-low-density lipoproteins, and lipid absorption in the intestine. To date, there have been two known cases of congenital diarrhea associated with mutations in *DGAT1*, which encodes acyl CoA:diacylglycerol acyltransferase 1, an enzyme that is involved in triglyceride synthesis and is highly expressed in the intestine (Fig. 4) (Haas et al., 2012). The mechanism by which *DGAT1* mutations cause diarrhea is unclear, but is likely to involve the build-up of DGAT1 lipid substrates in the enterocytes or in the gut lumen (Haas et al., 2012). *Dgat1* KO mice do not develop diarrhea, and it has been proposed that this is due to compensatory *Dgat2* expression in the mouse intestine (Buhman et al., 2002). The observation that the overexpression of Sar1b in human Caco-2 cells stimulated DGAT and MTTP activity (Levy et al., 2011) underscores the fact that all currently known CDD^ENT^ that are associated with defective lipid absorption originate in defects in the triglyceride-rich lipoprotein assembly pathway.

## Defects in intestinal barrier function

The barrier function of the intestine is important for fluid homeostasis and critically depends on cell-cell adhesions. Defects in the intestinal barrier function have been associated with at least one CDD.

### Congenital tufting enteropathy

Congenital tufting enteropathy (CTE) is characterized by persistent diarrhea that presents immediately or shortly after birth, despite bowel rest and total parenteral nutrition (TPN) (Goulet et al., 2007). Some affected individuals display a milder phenotype than others, and these can sometimes be progressively weaned off TPN (Lemale et al., 2011). A subset of individuals with CTE display a syndromic form of the disease [congenital sodium diarrhea (CSD)] that includes dysmorphic features, woolly hair, punctate keratitis, atresias, reduced body size and immune deficiency (see Box 1). Like THES, CTE can present as very-early-onset inflammatory bowel disease (Kammermeier et al., 2014).

**Fig. 5.**
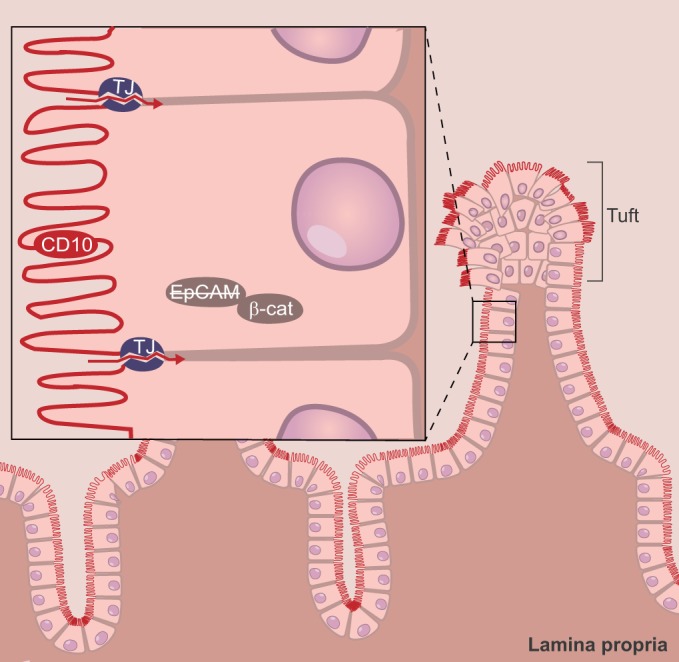
**Schematic overview of tissue and cellular defects associated with CTE.** In congenital tufting enteropathy (CTE), villi are shortened and are disorganized, with focal crowding of enterocytes (tufts). Mutated EpCAM is mislocalized intracellularly, which results, through an unknown mechanism, in the loss of tight junction (TJ) integrity and a concomitant increase in permeability (red arrows). TJ, tight junction; β-cat, β-catenin.

Histological analysis of the intestine in the context of CTE reveals various degrees of villous atrophy, basement membrane abnormalities, disorganization of enterocytes, and focal crowding at the villus tips, resembling tufts (Fig. 5). There is no evidence for abnormalities in epithelial cell polarization; the enterocyte brush border appears normal, and the staining pattern of the brush-border-associated metallopeptidase CD10 is normal (Martin et al., 2014), but expression of desmogleins, a family of cadherins, is enhanced (Goulet et al., 2007). The major diagnostic marker is the absence of epithelial cell adhesion molecule (EpCAM) staining in CTE enterocytes (Martin et al., 2014). Furthermore, immune cell infiltration into the lamina propria is absent. In some cases, however, increased numbers of inflammatory cells have been reported in the lamina propria, indicating that their presence does not preclude the diagnosis of CTE (Kammermeier et al., 2014).

CTE is associated with *EPCAM* or *SPINT2* mutations. EpCAM is a multifunctional transmembrane glycoprotein involved in cell-cell adhesion, proliferation and differentiation (Schnell et al., 2013b). In individuals with CTE, EpCAM protein levels in the intestine are decreased (Sivagnanam et al., 2008) and all CTE-associated *EPCAM* mutations lead to loss of cell-surface EpCAM (Fig. 5) (Schnell et al., 2013a), either because of impaired plasma membrane targeting or because of truncation of the protein, both of which result in its secretion. Both *Epcam* KO mice and mice in which exon 4 of *Epcam* is deleted develop CTE (Guerra et al., 2012; Kozan et al., 2015). In the *Epcam* KO mouse intestine, E-cadherin and β-catenin, two adherens-junction-associated proteins, are also mislocalized, leading to disorganized transition from crypts to villi (Guerra et al., 2012). Mice with reduced EpCAM levels and Caco-2 cells depleted of EpCAM show decreased expression of tight-junction proteins, increased permeability and decreased ion transport (Kozan et al., 2015). EpCAM interacts with the tight-junction proteins claudin-7 and claudin-1 (reviewed in Schnell et al., 2013b). Conceivably, loss of EpCAM expression and/or function leads to the increased permeability of the intestinal barrier by disrupting tight junctions (Fig. 5), resulting in diarrhea.

The mechanism by which mutations in *SPINT2* lead to CTE phenotypes, however, is not clear. *SPINT2* encodes the transmembrane Kunitz-type 2 serine-protease inhibitor. *Spint2* KO mice are embryonically lethal owing to developmental defects that are unrelated to the intestine (Szabo et al., 2009), and are therefore unsuitable for studying the intestinal symptoms of CTE. Interestingly, two of the target enzymes of Spint2 are the serine proteases matriptase and prostasin (Szabo et al., 2009), which are primary effector proteases of tight-junction assembly in intestinal epithelial cells (Buzza et al., 2010). The Y163C mutation in Spint2 results in a complete loss of the ability of Spint2 to inhibit prostasin and another intestinal protease, the transmembrane protease serine 13 (Tmprss13) (Faller et al., 2014). Further investigation is needed to determine the role of Spint2 and other proteases in the regulation of cell-cell junctions in the pathogenesis of CTE.

The inhibition of trypsin-family serine peptidases, such as that encoded by *SPINT2*, abolishes the constitutive stimulation of apical Na^+^ transport by nonvoltage-gated sodium channel-1-alpha (Scnn1a) in polarized intestinal epithelial cells (Planès and Caughey, 2007), which could contribute to secretory diarrhea. It is possible that such a mechanism forms the basis of the syndromic form of congenital sodium diarrhea that is associated with *SPINT2* mutations (Faller et al., 2014; Heinz-Erian et al., 2009).

Publically available bioinformatics gene co-expression databases show that the *EPCAM* and *SPINT2* genes are strongly co-expressed in humans (http://coxpresdb.jp/cgi-bin/coex_list.cgi?gene=4072&sp=Hsa, and http://coxpresdb.jp/cgi-bin/coex_list.cgi?gene=10653&sp=Hsa), which suggests that they either share a transcriptional regulatory program, are functionally related, or are members of the same pathway or protein complex. Interestingly, *ST14*, the gene that encodes matriptase, is strongly co-expressed with both *EPCAM* and *SPINT2*, further underscoring the need to study its involvement in the pathogenesis of CTE.

## Outlook and future perspectives

Establishing a molecular diagnosis for CDD^ENT^ is becoming feasible in most cases, and can be a key contributor to clinical decision making. At the moment, the prognosis and survival of individuals with CDD^ENT^ depend on early TPN and successful bowel transplantation, but survival is generally poor. A variety of extra-intestinal symptoms are associated with CDD^ENT^. Of these, renal Fanconi syndrome in MVID disappears after bowel transplantation (Golachowska et al., 2012), whereas intrahepatic cholestasis in MVID is aggravated after bowel transplantation (Girard et al., 2014; Halac et al., 2011). It remains unclear whether these symptoms are iatrogenic, i.e. complications of treatment, and/or are linked to particular CDD^ENT^-associated gene mutations or the genetic background of the patient. Prospective patient registries, animal models, and stem-cell-based organoid technology combined with novel gene-editing tools, such as CRISPR, will address these current shortcomings in our knowledge, as discussed below (see Box 3).

**Box 3. Clinical and basic research opportunities**

• The use of genetics and automated microscopy in the differential diagnosis of CDD^ENT^ in the clinic.

• The development of genetically engineered animal models of CDD^ENT^.

• The creation of CDD^ENT^ patient-specific stem-cell-based organoids for disease modeling.

• The establishment and maintenance of CDD^ENT^ patient registries that integrate basic and clinical data.

• The exploration of stem-cell-based replacement strategies as a potential cure for CDD^ENT^.

### Patient registries and databases

Dedicated patient registries are crucial resources for correlating the genotype, phenotype and clinical presentation of individuals with CDD^ENT^. Thus far, only a registry of patients with MVID and associated *MYO5B* mutations has been established (http://www.mvid-central.org) (van der Velde et al., 2013). Given that individuals with CDD^ENT^ display partially overlapping phenotypes, the expansion of such a database to include other CDD^ENT^ patients, including a prospective set-up that allows the course of disease to be recorded together with the influence of therapeutic interventions, is expected to improve disease diagnosis, prognosis and counseling.

### Vertebrate and invertebrate model organisms for CCD^ENT^

Intestinal epithelial cell lines cannot recapitulate all of the phenotypes associated with CDD^ENT^, such as those related to the different states of proliferation and differentiation in enterocytes as they migrate from the crypts to the villus tips in the intestine. This is important for understanding the cellular defects seen in MVID and CTE, which are more pronounced in the villus than they are in the crypt region (Groisman et al., 1993; Phillips et al., 2000; Thoeni et al., 2014). Cell lines also do not form villi, precluding the study of villi defects, villus atrophy and villus tufts. Finally, studies in intestinal cell lines do not take into account effects beyond the intestine.

Animal models offer a useful system for determining causal relationships between genes and CDD^ENT^, for investigating disease pathogenesis, and for evaluating treatment options preclinically. KO animals are useful for studying the function of the targeted gene and for modeling CDD^ENT^ individuals with homozygous mutations, and gene-editing techniques such as CRISPR-Cas can be used to introduce patient-relevant homozygous and compound heterozygous missense mutations both in animal and cell-line models.

The potential use of model organisms other than mice for CDD^ENT^ research has not been fully explored. Intestinal brush border proteins are normally apically localized in invertebrate nematode *Caenorhabditis elegans* worms that lack *Hum2*, the ortholog of *MYO5* (Winter et al., 2012). Conceivably, this reflects the distinct physiology and cellular architecture of the worm intestine. In developing larvae of the fly *Drosophila melanogaster*, myosin-V deficiency interferes with apical protein secretion in the hindgut (Massarwa et al., 2009). This suggests a problem with apical protein delivery and warrants further research to examine the potential of myosin-V-deficient flies as a model for CDD^ENT^. Other CDD^ENT^-associated genes have not yet been examined in worms or flies.

The ability to perform high-throughput assays and intravital imaging in vertebrate zebrafish (van Ham et al., 2014) make these animals a promising model for studying the effect of genetic manipulations and pharmacological treatment. Intestinal anatomy and architecture in zebrafish closely resemble the anatomy and architecture of the mammalian small intestine (Yang et al., 2014) and have been used to study enteropathies such as congenital short bowel syndrome (Van Der Werf et al., 2012). Zebrafish could therefore make a useful addition to current CDD^ENT^ models. Indeed, *sar1b*-deficient zebrafish display phenotypes resembling CMRD (Levic et al., 2015). The absence of the myosin-V ortholog in zebrafish results in an abnormal epidermal tissue structure. In the study reporting this mutant, inclusion bodies in the intestine are mentioned (Sonal et al., 2014). *epcam*-deficient zebrafish have aberrant epidermal development; however, intestinal defects have not been reported (Slanchev et al., 2009).

### Stem-cell-based organoids

Advances in stem cell technology provide new models for studying CDD^ENT^. Generating three-dimensional cultures of stem-cell-derived intestinal cells that resemble to some extent the intestinal tissue (so-called organoids) enables disease modeling that better resembles the *in vivo* situation while still retaining experimental versatility and the ability to genetically manipulate cells. Organoids allow for patient-specific personalized disease modeling. Promisingly, intestinal organoids generated from *STX3*-mutation-carrying individuals with MVID recapitulate most of the *in vivo* phenotypes (Wiegerinck et al., 2014).

Intestinal organoids can be generated from adult stem cells and by differentiating induced pluripotent stem cells (iPSCs) into intestinal cell types (Forster et al., 2014; Sato et al., 2009; Spence et al., 2011). Although both adult-stem-cell- and iPSC-derived intestinal tissue structures are referred to as organoids, notable differences exist between the two. Organoids obtained from iPSCs, but not from adult stem cells, contain supporting mesenchymal cells. Moreover, iPSC-derived organoids are relatively immature with fetal-like characteristics, although transplantation of iPSC-derived immature organoids under the kidney capsule of mice results in the development of mature, engrafted intestinal tissue that develops villi and crypts (Watson et al., 2014). From adult stem cells, only genomically engineered organoids that contain tumorigenic mutations have undergone successful engraftment under the mouse kidney capsule, suggesting that mesenchymal cells are required for organoid maturation outside of the intestinal niche (Matano et al., 2015). However, adult-stem-cell-derived organoids have been reported to engraft in the chemically injured mouse colon, to contribute to tissue regeneration, and to be indiscernible from host epithelium (Yui et al., 2012).

These differences are important to consider when organoids are used to study CDD^ENT^. The investigation of phenotypes that manifest at a multicellular level, such as the structural villi abnormalities in MVID and CTE, requires a model that forms villi and crypts. The maturity of organoids is also relevant because CDD^ENT^ phenotypes do not always manifest immediately after birth (e.g. late-onset forms). A practical consideration is that adult-stem-cell-derived organoid culture requires invasive biopsies, whereas the somatic cells to generate iPSCs can be non-invasively acquired.

Organoid technology uniquely allows the creation of patient-specific disease models. Despite harboring mutations in the same protein, many individuals with CDD^ENT^ often vary in the range and severity of their symptoms. This suggests that different mutations could have a varying effect on protein function, and thus on disease outcome. Other potential factors that could influence such variation are the genetic background of a patient and any adverse effects of treatment. Organoids from affected individuals with varying symptoms exclude confounding environmental factors and provide a model in which phenotypes are tissue-autonomous and solely dependent on patient genotype. The use of gene-editing tools, such as CRISPR, in organoid cultures could provide a valuable tool for making definitive genotype-phenotype correlations. Finally, organoids created from different organs of the same patient could provide additional insights into the genetic relationship of extra-intestinal symptoms associated with CCD^ENT^.

Although diagnostic tools for CCD^ENT^ have improved over the last few years, a cure for CDD^ENT^ is desperately needed. Organoid transplantation and/or cell-replacement strategies can lead to the restoration of the intestinal epithelium in mice (Yui et al., 2012). This raises the exciting possibility of investigating whether CRISPR-based correction of mutations in patient stem cells and transplantation of genetically corrected organoids could represent a regenerative medicine approach to cure CDD^ENT^.
